# Blood-based prognostic scores and early dynamics under immunotherapy to select patients with metastatic solid tumors for continuing immune check-point inhibition: a prospective longitudinal study

**DOI:** 10.1007/s00262-024-03933-w

**Published:** 2025-02-01

**Authors:** Javier García-Corbacho, Alberto Indacochea, Iván Victoria, Débora Moreno, Laura Angelats, Azucena E. González Navarro, Laura Mezquita, Fara Brasó-Maristany, Patricia Galván, Begoña Mellado, Nuria Viñolas, Tamara Sauri, Miquel Nogué, Barbara Adamo, Joan Maurel, Estela Pineda, Lydia Gaba, Oscar Reig, Neus Basté, Esther Sanfeliu, Manel Juan, Aleix Prat, Francesco Schettini

**Affiliations:** 1 The Clinical Trials Unit of Medical Oncology Department, Virgen de la Victoria University Hospital/IBIMA, Campus de Teatinos S/N, 29010 Malaga, Spain; 2 Programa de Doctorado en Biomedicina, Universitat de Barcelona, Barcelona, Spain; 3 Medical Oncology Department, Hospital Clinic of Barcelona, C. Enric Granados, 86-88, 08008 Barcelona, Spain; 4 Translational Genomics and Targeted Therapies in Solid Tumors, August Pi I Sunyer Biomedical Research Institute (IDIBAPS), Barcelona, Spain; 5 Medical Oncology Department, Hospital General of Granollers, Granollers (BCN), Spain; 6 Immunology Service, Hospital Clinic of Barcelona, Clinic Foundation for Biomedical Research - August Pi I Sunyer Biomedical Research Institute (FCRB-IDIBAPS), Barcelona, Spain; 7 Reveal Genomic, Barcelona, Spain; 8 Pathology Department, Diagnostic Biomedical Center, Hospital Clinic of Barcelona, Barcelona, Spain; 9 Faculty of Medicine and Health Sciences, University of Barcelona, Barcelona, Spain; 10 Institute of Cancer and Blood Diseases, Hospital Clinic of Barcelona, Barcelona, Spain; 11 Breast Cancer Unit, Institute of Oncology Barcelona (IOB) - Quirónsalud, Barcelona, Spain

**Keywords:** LIPI score, Immune check-point inhibitors, Immunotherapy, Metastatic, Cancer

## Abstract

**Introduction:**

Immune check-point inhibitors (ICI) were a major breakthrough in cancer care, but optimal patient selection remains elusive in most tumors.

**Methods:**

Overall 173 adult patients with metastatic solid tumors candidates to ICI in clinical trials at our Institution were prospectively recruited. Blood samples were collected at cycle 1 (C1D1) and 2 (C2D1) and until the occurrence of progressive disease (PD). C1D1 LIPI, RMH, PMHI, NLR, dNLR, PIPO and GRIm prognostic scores were calculated. The primary endpoint was identifying the best score to predict rapid PD (≤ 4 months) with ICI using logistic regressions accounting for tumor type, and receiving operators characteristics (ROC) with area under curve (AUC), accompanied by an extensive comparison of the score performances in the prediction of overall survival (OS), progression-free survival (PFS), overall response rates (ORR) and durable clinical benefit (DCB). Secondary objectives included describing study cohort outcomes and studying the association between the selected score at C1D1, C2D1 and its dynamics with OS and PFS.

**Results:**

C1D1 LIPI was the best predictor of rapid PD, OS and PFS, regardless of cancer type, compared to other scores. No score was associated to ORR and only RMH to DCB. Baseline LIPI detected three categories of patients with significantly different OS (*p* < 0.001) and PFS (*p* = 0.013). The same was observed at C2D1 for OS and PFS (both *p* = 0.020). Significant LIPI class shifts were observed in the overall population (*p* < 0.001), rapid progressors (*p* = 0.029) and non-rapid progressors (*p* = 0.009). Retaining a good LIPI or experiencing a shift towards a better prognostic class was associated to improved OS (*p* = 0.009) and PFS (*p* = 0.006). C2D1 LIPI, but not C1D1, remained significantly associated to rapid PD in multivariable analysis.

**Conclusions:**

LIPI may improve patient selection for ICI and guide treatment adjustments according to on-treatment dynamics in a pancancer context.

**Supplementary Information:**

The online version contains supplementary material available at 10.1007/s00262-024-03933-w.

## Introduction

Immunotherapy with immune check-point inhibitors (ICI) has represented a major breakthrough for the treatment of solid malignancies in the last decade. This therapeutic approach unleashes a potent immune response against the tumor by interfering with the activity of key molecules implied in the negative regulation of immune response [1]. However, ICI treatment efficacy is extremely heterogeneous and unpredictable, not only across different cancer histologies but also within a particular cancer type [2, 3]. Furthermore, ICIs may lead to harmful and potentially lethal immune-mediated side effects. Therefore, the identification of proper biomarkers of response is crucial to improve therapeutic outcomes, avoid unnecessary toxicities and optimize resources, since ICIs are considered an expensive treatment that can yearly cost more than $100,000 per patient [4]. Unfortunately, few biomarkers have proved to be effective for a proper patient selection and with several cancer-specific and/or technical-related limitations [5–7].

With the aim of characterizing clinical features, tissue-based and blood-based biomarkers that could predict prognosis and response to ICI in a pancancer context, we carried out the Bioimmunoblood prospective observational study at the Clinical Trials Unit (CTU) of the Hospital Clinic of Barcelona (HCB) [8]. In this report, we assessed clinical outcomes in the entire study cohort, compared the most relevant baseline prognostic scores developed for selecting or stratifying candidates to ICI and/or phase I trial across different tumor types [9–18] to pick the best one in predicting rapid progression to ICI, and further assess the contribution of the most successful score’s early dynamics to detect rapid progressors and improve patient selection for continuing immune check-point inhibition.

## Methods

### Study design and participants

To participate in the Bioimmunoblood study, eligible patients had an advanced solid cancer and a scheduled initiation of an ICI-based treatment in a clinical trial. Full inclusion/exclusion criteria were previously reported [8]. Patients were recruited between November 2016 and March 2022 and followed-up until December 2023. We considered evaluable for this analysis all participants treated with an ICI with radiological data available for an independent assessment of tumor responses according to the tumor type.

### Procedures

A blood sample was collected from each patient at the first day of cycle 1 (C1D1) and 2 (C2D1) prior to receive the treatment and, subsequently, at each radiological evaluation until progressive disease (PD) was determined [8]. For the purpose of the present analysis only blood samples at C1D1 and C2D1 were interrogated. Blood chemistry tests were carried out, including the evaluation of albumin, hemoglobin (Hb), lactate dehydrogenase (LDH) and standard leukocyte populations. The use of antibiotics (ATB) and/or corticoids during ICI was assessed. C1D1 samples were used for the election of the best prognostic predictor among the most commonly used, namely the Lung Immune Prognostic Index (LIPI), Royal Marsden Hospital (RMH), Princess Margaret Hospital Index (PMHI), neutrophil-to-lymphocyte ratio (NLR), derived NLR (dNLR), Phase I Prognostic Online (PIPO) and Gustave Roussy Immune (GRIm) prognostic scores [9–18]. C2D1 samples were also analyzed to assess the best score’s dynamics between C1D1 and C2D1. Treatment decisions were made outside of this study according to trial protocol and investigators criteria as ICI-based treatments were given as part of the clinical trials conducted at the CTU of the HCB. All data were retrieved from electronic patient charts.

### Study endpoints and outcomes

There was no prespecified sample size because of the exploratory nature of this study. The clinical data cut-off was established when a minimum follow-up including at least one reassessment of the disease for every included patient was reached.

The primary objective of this analysis was to provide an extensive comparison of the prognostic scores in terms of prediction of overall survival (OS), progression-free survival (PFS), overall response rates (ORR) and durable clinical benefit (DCB) with ICI, and identify the best score predicting rapid PD to ICI. The key secondary objective was a more refined assessment of the association between the selected best score at C1D1 with OS, PFS, ORR and DBC by accounting for relevant confounding factors. Other secondary objectives were the assessment of the association between the selected score at C2D1 with PFS and at C2D1 with OS, the evaluation of the best score’s category changes from C1D1 to C2D1 as well as the evaluation of the association of the best score’s dynamics with PFS and OS.

**Fig. 1:**
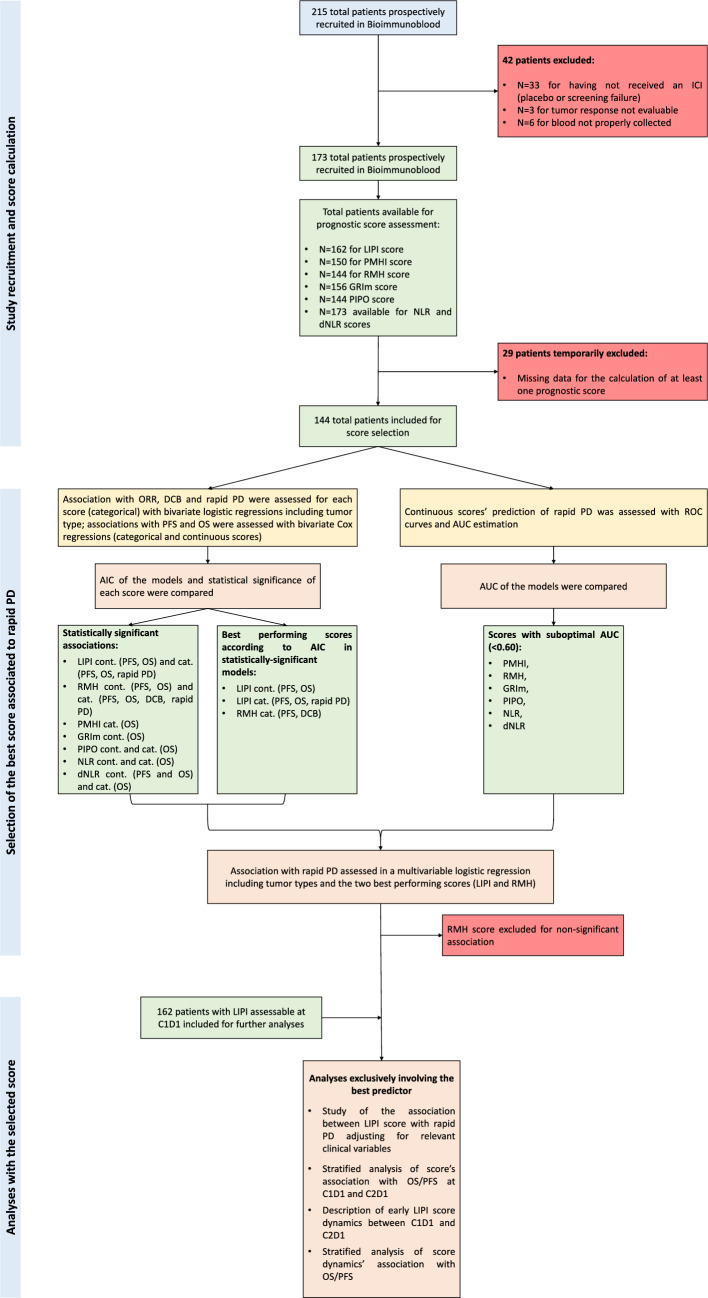
Study description. AIC: Akaike information criterion; AUC: ROC’s area under curve; C: treatment cycle; D: day; dNLR: derived NLR; GRIm: Gustave Roussy Immune prognostic score; N: number of patients; NLR: neutrophil-to-lymphocyte ratio; LIPI: Lung Immune Prognostic Index; OS: overall survival; PD: progression of the disease; PFS: progression-free survival; PMHI: Princess Margaret Hospital Index; PIPO: Phase I Prognostic Online; RMH: Royal Marsden Hospital; ROC: receiving operator characteristics

PFS was defined as the time from C1D1 to PD or death from any cause, whichever occurred first. OS was defined as the time from C1D1 to death from any cause. Rapid PD was defined as PFS ≤ 4 months from ICI initiation. This cut-off was determined by the study authors as the minimum clinically acceptable benefit achievable with ICIs, taking into account the balance between the potential treatment toxicity, costs, potential need to detect pseudo-progressions and the prognosis of advanced solid tumors [19]. The evaluation of response was performed in accordance to RECIST 1.1 criteria [20], or RANO criteria in the case of glioblastomas (GB) [21]. Best overall responses (BOR) were classified as PD, stable disease (SD), complete (CR) or partial response (PR) by the same expert (Dr. García-Corbacho). ORR included all patients achieving CR or PR as BOR. DCB was defined as absence of PD at 6 months [8]. The score selection process and main study analyses are resumed in Fig. 1.

### Statistical analysis

When appropriate, χ^2^, Kruskal–Wallis and Wilcoxon rank-sum tests, for unpaired variables, and McNemar and Wilcoxon signed-rank tests, for paired variables, were used to calculate differences between patients with different prognostic score classes and rapid vs. non-rapid PD. Bivariate logistic regression analyses were performed to estimate the odds ratios (OR) with their 95% confidence intervals (CI) to investigate the association of the prognostic scores at C1D1 with rapid PD, ORR and DCB. Bivariate Cox proportional hazard models including tumor type and each score at C1D1 were used to estimate hazard ratios (HR) with their 95% CI to explore associations with PFS and OS. Patients alive were censored at the date of the last follow-up. Akaike information criterion (AIC) was used to compare the goodness of fit among multivariable regression models [22]. A difference of more than 2 points between models was considered significant, and the model with the lowest AIC was considered to be the best [23]. Receiving operator characteristics (ROC) curves and their area under curve (AUC) were then used for each score to assess the capability of predicting rapid PD. AUCs were then compared with the DeLong test. After identifying the score with the best performance on all endpoints and the best capability of predicting rapid PD, Cox proportional hazard models stratified for selected confounders were then used to explore the association of the best score at C1D1, C2D1 and score dynamics between C1D1-C2D1 with PFS and OS (Supplementary methods). Multivariable logistic regressions accounting for the same confounders were carried out to assess the best score at C1D1, C2D1 and C1D1-C2D1 dynamics with rapid PD. The proportional hazard assumption was properly checked [24] for both OS and PFS (Supplementary Fig. 1). Survival curves were estimated by the Kaplan–Meier method and differences between curves were evaluated by the log-rank test. Landmark analyses to assess 12-month and 24-month OS and PFS according to the best score classes were conducted, as well. No imputation was done for missing data. A two-sided alfa error of 0.05 was considered for statistical significance.

All statistical analyses were carried out using R Studio vers.1.0.153 (PBC, Boston, MA) and SPSS vers. 24.0 (IBM SPSS Statistics, Armonk, NY: IBM Corp) for MacOSX.

## Results

### Population characteristics, risk stratification and outcomes

**Fig. 2:**
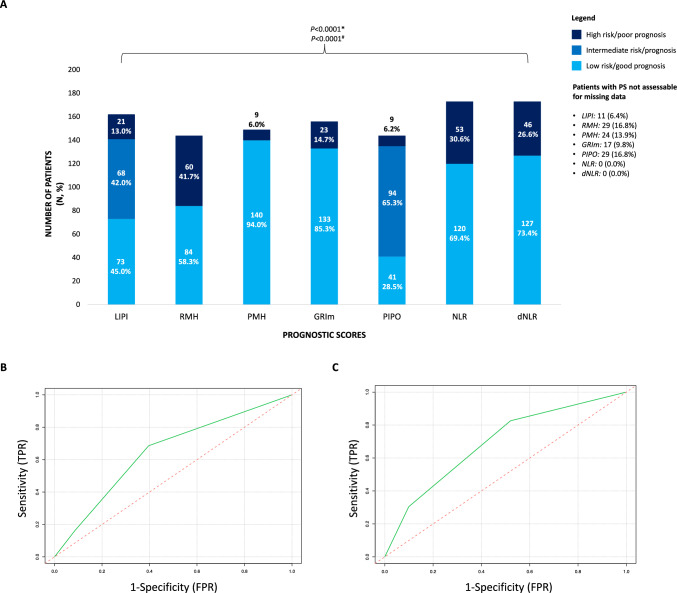
Prognostic stratification of Bioimmunoblood patients according to different prognostic scores and ROC curves of LIPI score as a predictor of rapid PD. **A** Prognostic stratification of study population according to different prognostic scores at baseline; **B** ROC curve of the LIPI score as a predictor of rapid PD; **C** ROC curve of the LIPI score as a predictor of rapid death, excluding disease progression without survival events. dNLR: derived NLR; GRIm: Gustave Roussy Immune prognostic score LIPI: Lung Immune Prognostic Index; NLR: neutrophil-to-lymphocyte ratio; PD: progression of the disease; PIPO: Phase I Prognostic Online; PMHI: Princess Margaret Hospital Index; RMH: Royal Marsden Hospital; ROC: receiving operator characteristics. % were calculated according to the total number of patients with available parameters to assess each prognostic score. Missing values for each score are reported in the in-figure legend. *: for this analysis the intermediate prognostic group of the LIPI and PIPO score was jointed with the respective poor prognostic group. #: for this analysis the intermediate prognostic group of the LIPI and PIPO score was jointed with the respective good prognostic group

A total of 173 patients were included. Population demographics at baseline are reported in Table 1. The baseline prognostic stratification provided by the selected prognostic scores is reported in Fig. 2A. The proportion of patients pertaining to the same prognostic category according to the different predictors varied significantly (*p* < 0.001).

**Table 1:** Bioimmunoblood population characteristics

Demographics	Overall	
	N	%
	173	100.0
Age		
Median	64	–
IQR	56.8—71.5	–
Sex		
Male	108	62.4
Female	65	37.6
*Overall*	*173*	*100.0*
ECOG		
0–1	153	88.4
≥ 2	20	11.6
*Overall*	*173*	*100.0*
Tumor type		
Breast cancer	15	8.7
Colorectal adenocarcinoma	33	19.1
NSCLC	47	27.2
Head and neck	10	5.8
Gynecologic tumors (Cervix, endometrium, ovary)	9	5.2
Pancreas and biliary tract tumors	6	3.5
Esophageal and gastric carcinoma	9	5.2
Melanoma	9	5.2
Prostate adenocarcinoma	8	4.6
Renal cell carcinoma	7	4.0
Urothelial bladder cancer	6	3.5
Glioblastoma	8	4.6
Other*	6	3.5
*Overall*	*173*	*100.0*
Number of metastatic sites		
< 3	34	19.7
≥ 3	139	80.3
*Overall*	*173*	*100.0*
Metastatic involvement		
Visceral	139	80.3
CNS§	10	5.8
*Overall*	*173*	*100.0*
RT ≤ 30 days from ICI start		
Yes	10	5.8
No	162	94.2
*Overall*	*172*	*99.4*
Systemic ATB ≤ 30 days from ICI start		
Yes	9	5.2
No	164	94.8
*Overall*	*173*	*100.0*
Systemic ATB during ICI		
Yes	53	30.6
No	120	69.4
*Overall*	*173*	*100.0*
Systemic corticosteroids ≤ 30 days from ICI start		
Yes	23	13.3
No	150	86.7
*Overall*	*173*	*100.0*
Systemic corticosteroids during ICI		
Yes	67	38.7
No	106	61.3
*Overall*	*173*	*100.0*
ICI treatment line		
1st	46	26.6
2nd	54	31.2
≥ 3rd	73	42.2
*Overall*	*173*	*100.0*
ICI type		
Anti-PD1	131	75.7
Anti-PD-L1	32	18.5
Other	10	5.8
*Overall*	*173*	*100.0*
Regimen type		
ICI monotherapy	92	53.2
ICI combination	36	20.8
ICI + other agent	45	26.0
*Overall*	*173*	*100.0*
Previous immunotherapy in every setting		
Yes	139	80.3
No	34	19.7
*Overall*	*173*	*100.0*

PS: performance status; IQR: interquartile range; NSCLC: non-small cell lung cancer; ATB: antibiotics; RT: radiotherapy; CNS: central nervous system; ICI: immune-checkpoint inhibitor. *: thymic carcinoma, Merkel cell carcinoma, carcinomas of unknown primary site, soft tissue sarcomas, adrenal gland adenocarcinoma, hepatocarcinoma; #: 2 patients received ICI in 1st or 2nd line, but the precise information was not reported in our records; §: excluding glioblastomas

At a median follow-up of 38.3 months (95%CI 36.1–58.6), median PFS (mPFS) was 2.5 months (95%CI 2.0–3.7) and median OS (mOS) was 13.3 months (95%CI 9.9–17.4), with rapid PD or death ≤ 4 months from ICI initiation observed in 60.7% (95%CI 53.0–68.0%) cases. Objective responses were scarce, with an ORR of 16.8% (95%CI 11.5–23.2%). Main outcomes are detailed in Table 2.

**Table 2:** Activity and efficacy of ICI in the overall study population

Study population outcomes		
*PD timing*	*N*	*%*
≤ 4 months from ICI start	105	60.7
> 4 months from ICI start	68	39.3
*Best response*	*N*	*%*
Complete response	7	4.0
Partial response	22	12.7
Stable disease	59	34.1
Progressive disease	85	49.1
*ORR*	*%*	*95%CI*
CR + PR	16.8	11.5%—23.2%
DCB	*%*	*95%CI*
CR + PR + SD ≥ 6 months	30.1	23.2% – 36.9%
*Median PFS (months)*	*N*	*95%CI*
	2.5	2.0—3.7
*Median OS (months)*	*N*	*95%CI*
	13.3	9.9—17.4
*12-month PFS*	*N (%)*	*95%CI*
	32 (18.5)	13.5%—25.3%
*12-month OS*	*N (%)*	*95%CI*
	88 (50.9)	45.0%—60.0%

CI: confidence interval; CR: complete response; DCB: durable clinical benefit; ICI: immune-checkpoint inhibitor; N: number; ORR: overall response rate; PD: disease progression; PR: partial response; PFS: progression-free survival; SD: stable disease; OS: overall survival

### Identification of the best score predicting PFS, OS, ORR, DCB and rapid PD

We subdivided the study cohort into rapid progressors (RP) (PFS ≤ 4 months from ICI initiation) and non-rapid progressors (NRP) (PFS > 4 months from ICI initiation). The groups differed significantly in several baseline clinicopathological factors, as reported in Table 3. We explored which was the best prognostic predictor among LIPI, RMH, PMHI, NLR, dNLR, PIPO and GRIm scores in terms of PFS and OS. First, we removed from this analysis all patient with at least one of the scores not assessable, in order to avoid a biased comparison between scores due to their different populations. Following this selection we reached a total population of 144 patients. We run multiple bivariate Cox regression models each one including one of the scores as continuous or categorical variable and tumor types. Only LIPI (*p* = 0.001), dNLR (*p* = 0.024) and RMH (*p* = 0.006) continuous scores, and LIPI (*p* = 0.002) and RMH (*p* = 0.001) categorical scores were significantly associated to PFS, regardless of tumor type (Supplementary Table 1). The best AIC was observed for the model including continuous LIPI score (AIC: 1085.70). Similar AIC was observed for the models including categorical LIPI (AIC: 962.59) and RMH (AIC: 968.62). LIPI (*p* < 0.001), NLR (*p* = 0.025), dNLR (*p* < 0.001), RMH (*p* = 0.005), GRIm (*p* = 0.006) and PIPO (*p* = 0.030) continuous scores were significantly associated to OS independently of tumor type. Similarly, LIPI (*p* < 0.001), NLR (*p* = 0.023), dNLR (*p* < 0.001), RMH (p < 0.001), PMHI (*p* = 0.046) and PIPO (*p* = 0.033) categorical scores were significantly associated to OS (Supplementary Table 1). The bivariate model including LIPI showed the best AIC both for the continuous (AIC: 961.12) and categorical (AIC: 962.59) score. No score was significantly associated to ORR (not shown) and only RMH was associated to DCB (adjusted OR [aOR] for high score vs. low: 0.34, 95%CI: 0.14-0.83, *p* =0.017).

**Table 3:** Baseline clinicopathological features according to rapid tumor progression status

Demographics	Rapid progressors		Non-rapid progressors		*P*
	N	%	N	%	
	105	60.7	68	39.3	
*Age*					
Median	62.6	–	67.1	–	*0.030*
IQR	54.2—69.0	–	60.5—72.6	–	
*Sex*					
Male	61	58.1	47	69.1	0.144
Female	44	41.9	21	30.9	
*Overall*	105	100.0	68	100.0	
*ECOG*					
0–1	94	89.5	59	86.8	0.579
≥ 2	11	10.5	9	13.2	
*Overall*	105	100.0	68	100.0	
*Tumor type*					
Breast cancer	12	11.4	3	4.4	*0.031*
Colorectal adenocarcinoma	23	21.9	10	14.7	
NSCLC	22	21.0	25	36.8	
Head and neck	7	6.7	3	4.4	
Gynecologic tumors (Cervix, endometrium, ovary)	5	4.8	4	5.9	
Pancreas and biliary tract tumors	4	3.8	2	2.9	
Esophageal and gastric carcinoma	6	5.7	3	4.4	
Melanoma	7	6.7	2	2.9	
Prostate adenocarcinoma	0	0.0	8	11.8	
Renal cell carcinoma	4	3.8	2	2.9	
Urothelial bladder cancer	5	4.8	2	2.9	
Glioblastoma	6	5.7	2	2.9	
Other*	4	3.8	2	2.9	
*Overall*	105	100.0	68	100.0	
*Number of metastatic sites*					
< 3	21	20.0	13	19.1	0.887
≥ 3	84	80.0	55	80.9	
*Overall*	105	100.0	68	100.0	
*Metastatic involvement*					
Visceral	86	81.9	53	77.9	0.522
Non-visceral	19	18.1	15	22.1	
*Overall*	105	100.0	68		
CNS metastases	6	6.1	4	6.1	1.000
No CNS metastases	93	93.9	62	93.9	
*Overall§*	99	94.3	66	97.1	
*RT* ≤ *30 days from ICI start*					
Yes	6	5.8	4	5.9	0.975
No	98	94.2	64	94.1	
*Overall*	104	99.0	68	100.0	
*Systemic ATB* ≤ *30 days from ICI start*					
Yes	5	4.8	4	5.9	0.746
No	100	95.2	64	94.1	
*Overall*	105	100.0	68	100.0	
*Systemic ATB during ICI*					
Yes	24	22.9	29	42.6	*0.006*
No	81	77.1	39	57.4	
*Overall*	105	100.0	68	100.0	
*Systemic corticosteroids* ≤ *30 days from ICI start*					
Yes	13	12.4	10	14.7	0.660
No	92	87.6	58	85.3	
*Overall*	105	100.0	68	100.0	
*Systemic corticosteroids during ICI*					
Yes	28	26.7	39	57.4	< *0.001*
No	77	73.3	29	42.6	
*Overall*	105	100.0	68	100.0	
*ICI treatment line*					
1st	21	20.0	25	36.8	*0.044*^*#*^
2nd	33	31.4	20	29.4	
≥ 3rd	51	48.6	23	33.8	
*Overall*	105	100.0	68	100.0	
*ICI type*					
Anti-PD1	78	74.3	54	79.4	*0.044*
Anti-PD-L1	18	17.1	14	20.6	
Other	9	8.6	0	0.0	
*Overall*	105	100.0	68	100.0	
*Regimen type*					
ICI monotherapy	54	51.4	38	55.9	*0.043*
ICI combination	28	26.7	8	11.8	
ICI + other agent	23	21.9	22	32.4	
*Overall*	105	100.0	68	100.0	
*Previous immunotherapy*					
Yes	81	77.1	58	85.3	0.188
No	24	22.9	10	14.7	
*Overall*	105	100.0	68	100.0	

ICI: immune-checkpoint inhibitors; ATB: antibiotics; NSCLC: non-small cell lung cancer; CNS: central nervous system; IQR: interquartile range. *thymic carcinoma, Merkel cell carcinoma, COD, STS, adrenal gland adenocarcinoma, hepatocarcinoma; §: excluding glioblastomas; #: chi-square for variable dichotomized in 1st and ≥ 2nd line

We then assessed the association of all categorical scores with rapid PD in bivariate logistic regression models including tumor types. A significant and independent association was observed with LIPI categorical score (*p* = 0.003); specifically, the poor vs. good (aOR: 6.00, 95%CI 1.47–24.53, *p* = 0.013) and the intermediate vs. the good categories (aOR: 3.36, 95%CI 1.48–7.62, *p* = 0.004), without significant differences between the poor and intermediate score categories (*p* = 0.422). RMH categorical score was also associated to rapid PD (aOR: 2.88, 95%CI 1.29–6.46, *p* = 0.010), but the best AIC was observed for the LIPI score-containing model (AIC: 192.69 and 189.31, respectively). All other scores were not significantly associated to rapid PD (not shown). All statistically significant models’ AIC for each endpoint are reported in Supplementary results.

The majority of the scores were unable to predict rapid PD at the ROC curve analysis, with an AUC ranging 0.51–0.60. LIPI score was the only one significantly able to predict rapid PD, with satisfactory efficacy (AUC: 0.65, 95%CI 0.56–0.74, DeLong *p* < 0.001) (Fig. 2B). The sensitivity and specificity were 68.6% and 60.3%, respectively, for a score class intermediate or poor to be able to detect RP, meaning that out of 100 true RP, 69 could be correctly identified and out of 100 NRP, 60 were correctly detected. Notably, the performance of LIPI score improved when early deaths were the only events considered (AUC: 0.69, 95%CI 0.56–0.82) (Fig. 2C).

Finally, we built a multivariable model to predict rapid PD including LIPI and RMH categorical scores and tumor types. Only LIPI score retained a significant association with rapid PD (poor/intermediate vs. good score category aOR: 3.10, 95%CI 1.12–8.60 p = 0.029).

Considering the globally superior performance of LIPI score in the previous assessments, it was ultimately selected for further analyses of its dynamics and more refined study of the associations with survival outcomes. To note, LIPI score at C1D1 was available for 162 (93.6%) patients. Baseline clinicopathological features according to LIPI score class are reported in Supplementary Table 2.

### Association between LIPI at C1D1 and OS/PFS

**Fig. 3:**
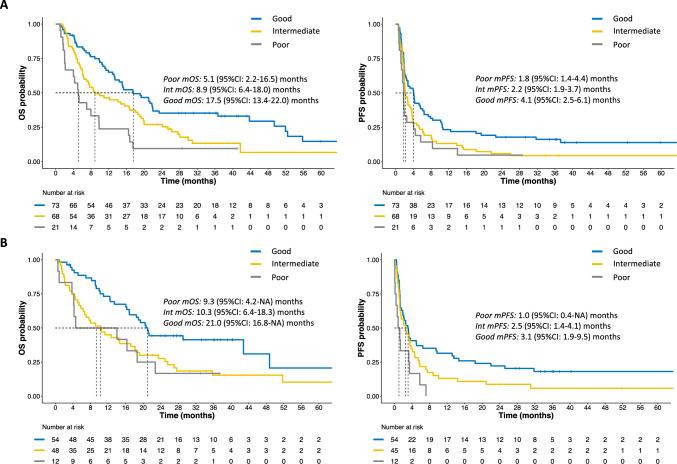
Kaplan–Meier curves of OS and PFS according to LIPI score at C1D1 and C2D1. **A** OS and PFS curves of LIPI at C1D1; **B** OS and PFS curves of LIPI at C2D1. CI confidence interval; C: cycle; D: day; Int: LIPI score intermediate class; m: median; OS: overall survival; PFS: progression-free survival. Good, Intermediate and Poor are referred to LIPI score classes

C1D1 LIPI score detected three categories of patients with significantly different OS (p < 0.001), where patients with a poor (stratified HR [HR_st_]: 2.74, 95%CI 1.56–4.80) and intermediate score (HR_st_: 1.63, 95%CI 1.09–2.44) showed significantly worse OS than patients with a good score (Fig. 3A) (stratified model’s AIC: 719.41). Similarly, LIPI score was also prognostic in terms of PFS (p = 0.013). At C1D1, the poor/intermediate scores (HR_st_: 1.55, 95%CI 1.10–2.18) score were associated with worse PFS than the good score (Fig. 3A) (stratified model’s AIC: 807.88). Landmark analyses of 12-month and 24-month OS and PFS provided consistent results (Supplementary Table 3).

### Association between LIPI at C2D1 and OS/PFS

LIPI at C2D1 was assessable for 114 (65.9%) patients. When considering LIPI score at C2D1 and stratifying also for LIPI at C1D1, a global significant difference in OS between the three score classes was observed (p = 0.020) (stratified model’s AIC: 60.57). The intermediate (HR_st_: 8.34, 95%CI 1.03–67.42) and poor (HR_st_: 40.75, 95%CI 1.59–1045.04) scores performed worse than the good (Fig. 3B). In terms of PFS, the intermediate (HR_st_: 1.64, 95%CI 1.01–2.67) and poor (HR_st_: 2.61, 95%CI 1.24–5.51) score classes showed a significantly worse outcome than the good score class (p = 0.020), stratifying also for baseline LIPI (Fig. 3B) (stratified model’s AIC: 453.49). Landmark analyses of 12-month and 24-month OS and PFS provided consistent results (Supplementary Table 3).

### LIPI score dynamics and association with rapid PD, OS and PFS

**Fig. 4:**
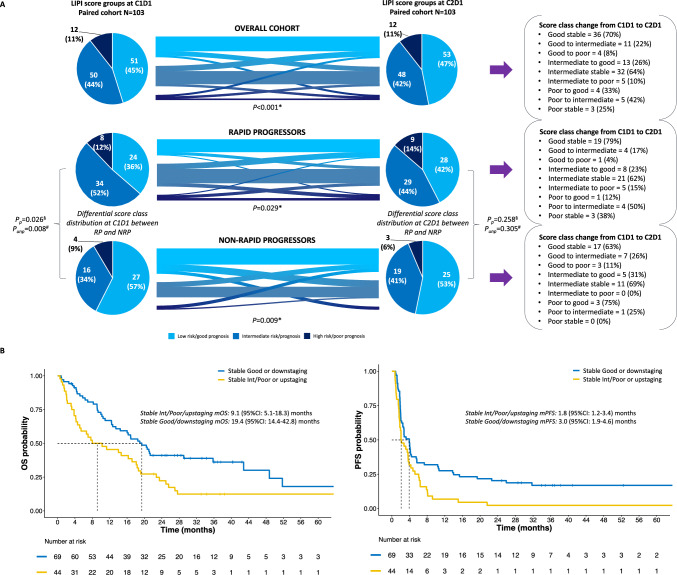
LIPI score early dynamics and association with survival outcomes. **A** LIPI score distribution at C1D1 and C2D1 and dynamics. **B** OS and PFS curves of LIPI dynamics between C1D1 and C2D1. CI: confidence interval; Int: LIPI score intermediate class; m: median; NRP: non-rapid progressors; OS: overall survival; PD: progression of the disease; PFS: progression-free survival, P_p_: p-values for the cohort with available C1D1 and C2D1 paired samples; P_unp_: *p* values for the total cohort with LIPI scores available at C1D1 and/or C2D1; RP: rapid progressors; #: *p* values from χ^2^ tests comparing LIPI good vs. LIPI intermediate/poor between RP and NRP; §*p* values from χ^2^ tests comparing LIPI good vs. intermediate vs. poor between RP and NRP; *p values from McNemar tests to assess LIPI dynamics in paired samples. Good, Intermediate and Poor are referred to LIPI score classes

Paired LIPI scores at C1D1 and C2D1 were assessable for 113 (65.3%) patients. We explored potential LIPI score changes from C1D1 to C2D1. A significant number of patients changed their LIPI class from C1D1 to C2D1. It happened in the overall population (*p* < 0.001), RP (*p* = 0.029) and NRP (*p* = 0.009). Despite those changes, the proportion of good, intermediate and poor classes between the two timepoints remained similar (Fig. 4A). When separating the study population into RP vs. NRP, by taking into account all patients (not only those with paired C1D1-C2D1 samples), we observed that NRP had 38 (60.3%) good, 19 (30.2%) intermediate and 6 (9.5%) poor LIPI score class cases, as compared to 35 (35.4%) good, 49 (49.5%) intermediate and 15 (15.1%) poor score cases in RP at C1D1 (*p* = 0.008). Conversely, no significant difference was observed at C2D1 (*p* = 0.305). Similarly, when restricting the comparison only to NRP and RP patients with paired LIPI score at C1D1 and C2D1, a significantly higher proportion of LIPI good cases for NRP vs. RP was still observed, with more intermediate/poor cases in the RP group (*p* = 0.026) and a similar proportion of LIPI score classes at C2D1 (*p* = 0.258) (Fig. 4A). Similarly to what previously done for LIPI at C1D1, we built a bivariate logistic regression model adjusting for tumor type, so to test the association of LIPI at C2D1 with rapid PD. A significant association was observed, as well (*p* = 0.049), especially for the poor vs. good score (aOR: 9.91, 95%CI 1.58–62.04) and the poor vs. intermediate (aOR: 6.83, 95%CI 1.06–43.87). Other comparisons did not reach statistical significance. A shift towards poorer score classes or stability of the intermediate and poor classes was not formally associated to rapid PD (OR: 2.28, *p* = 0.096).

When assessing the association of rapid PD with either LIPI at C1D1 or LIPI at C2D1 in a multivariable model accounting for all clinicopathological features differently distributed between RP and NRP (Table 3), as well as ECOG performance status (PS), LIPI at C1D1 was no longer associated (*p* = 0.593) whereas LIPI at C2D1 was. More specifically, the poor score vs. the good (OR_adj_: 6.95, 95%CI 1.19–40.51, *p* = 0.031) or intermediate (OR_adj_: 9.63, 95%CI 1.47–63.22, *p* = 0.018) scores retained a strong significant association with rapid PD, independently of baseline LIPI, age, tumor type, treatment line at which the ICI was delivered, ICI regimen type, the ICI target and the use of systemic ATB or corticosteroids during ICI treatment and ECOG. In addition, the multivariable model including both LIPI at C1D1 and C2D1 presented an AIC of 135.16, similar to the AIC of 133.07 exhibited by the model including only LIPI at C2D1 (not shown), while the model including only LIPI at C1D1 (not shown) showed an AIC of 191.65, supporting the value of assessing early LIPI dynamics to predict rapid PD.

In terms of prognosis, retaining a good prognostic score class between the C1D1 and C2D1 or experiencing a downstaging from poor to intermediate or good, or from intermediate to good was associated to significantly better OS (HR_st_: 1.90, 95%CI 1.17–3.09, *p* = 0.009) than remaining in the same intermediate or poor class or experiencing an upstaging (Fig. 4B and Supplementary Fig. 2) (stratified model’s AIC: 394.08). Consistently, in terms of PFS, those retaining a good prognostic score class between C1D1 and C2D1 or experiencing a downstaging experienced significantly better outcomes than all other cases (HR_st_: 1.86, 95%CI 1.18–2.92, *p* = 0.006) (Fig. 4B and Supplementary Fig. 2) (stratified model’s AIC: 452.14). We checked also if numerical score variations between the first two ICI treatment cycles, without considering the score class, could impact on prognosis. We did not observe any meaningful difference in PFS or OS (not shown).

## Discussion

In this prospective cohort of patients with advanced solid tumors treated with ICI in a clinical trial at our Institution, almost 61% of patients progressed or died within 4 months from ICI initiation, with roughly 17% achieving an objective response and 18% completed at least one year on ICI treatment. These findings are in line with current evidence about immunotherapy outcomes [25, 26]. Several prognostic scores have been developed to improve ICI and/or phase I clinical trial candidates selection [9–18]. Nevertheless, these scores’ capability of detecting rapid PD under ICI has not been tested and they have never been directly compared to predict objective responses and prognosis. Here, we performed a direct comparison of the most common scores in terms of PFS, OS, ORR, DCB and assessed the best predictor of a meaningful clinical benefit with ICI, considered to be a progression-free interval of more than 4 months. LIPI proved to be the best prognostic score, with the best performance in the identification of rapid progressions, with a solid association with OS and PFS beyond tumor type and relevant clinical features. In addition, LIPI score early dynamics between C1D1 and C2D1 were investigated and seemed to help improving prognostic prediction in terms of both PFS and OS beyond baseline score. Interestingly, the dNLR at baseline combined with its determination at C2D1 improved prediction of ICI outcomes in the context of advanced NSCLC, further suggesting that the early assessment of inflammatory biomarker dynamics can be a valuable prognostic tool beyond baseline determination [27].

At present, only PD-L1 positivity, high microsatellite instability (MSI-H)/mismatch repair deficiency (dMMR) and high tumor mutational burden (TMB-H) are clinically-approved biomarkers for ICI patient selection [5–7]. However, all of these biomarkers present several limitations, including low frequency [28], heterogeneity among cancer types [6, 29–31] and costs. Other immunohistochemical or transcriptomic-based biomarkers are currently under investigation by our group and others (e.g. PD1 mRNA levels, IGG signature, tumor-infiltrating lymphocytes, tertiary lymphoid structures, LORIS) [8, 32–39]. Nonetheless, they are still far from reaching the clinical practice scenario. In this context, LIPI score emerges as a cheap and easy-to-detect blood-based biomarker which effectively stratified patients in three prognostic groups, independently of main clinicopathological factors. This score, assessed by detecting blood levels of LDH and dNLR (absolute neutrophil count/[white blood cell concentration − absolute neutrophil count]) [16], accounts for peripheral pro-inflammatory status[40–42], a known poor prognostic factor in patients with cancer [42, 43]. Higher categories of LIPI score are the reflection of higher LDH and dNLR blood levels, suggesting more peripheral chronic inflammation. Our experience is coherent with the results of a large meta-analysis involving almost 10,000 patients in 35 studies, where LIPI score robustly stratified patients receiving ICI into three groups with different survival outcomes [44].

Focusing on rapid progressions, we considered 4 months as an acceptable cut-off to detect a minimum clinically meaningful benefit by ICI treatment, taking into account the need for radiologic re-assessments for pseudo-progressions, toxicity, costs and life expectancy of these pretreated population (40% received the ICI in ≥ 3rd line and several prognostically unfavorable cancers included). LIPI score, with a sensitivity of 68.6%, was reasonably good at detecting RP, differently from all other tested scores, also independently of cancer type and relevant clinical factors differentially distributed between RP and NRP, as well as ECOG PS. To note, when accounting for the same clinical factors along with baseline LIPI, LIPI at C2D1 showed a strong and independent prediction of rapid PD beyond the baseline score, especially for what concerns the poor score category. In fact, a poor LIPI at C2D1 was associated to an 595% and a 863% increase in the odds of achieving rapid PD in comparison to a good or intermediate score. Furthermore, when stratifying for relevant clinical factors and LIPI score at C1D1, the Cox model including C2D1 showed a higher goodness of fit than the stratified Cox model including only baseline LIPI, suggesting a more refined prognostic accuracy, as also supported by landmark analysis of 12- and 24-month PFS/OS.

From a biological perspective, a downstaging in LIPI score category might be related to either a reduction in LDH and/or dNLR, which is associated to a lowering of neutrophils and/or an increase in lymphocytes levels. On one hand, LDH has been classically linked to tumor burden and cancer metabolism, but important immunosuppressive effects were also described in more recent years [45, 46]. Thus, its reduction might both directly reflect tumor response to treatment, as well as a reduction in immunosuppression, favoring response to ICI. On the other hand dNLR reduction due to lymphocytes increase suggests an effective ICI-promoted activation of adaptive immune response against the tumor [47]. This combination of factors could thus explain not only the baseline prognostic role of the score, but also the association of its dynamics and levels at C2D1 with better long-term outcomes and less rapid progressions. Importantly, when we assessed the prognostic impact of score dynamics by considering the numerical variations instead of LIPI score class change, we did not detect any meaningful impact on prognosis. This leads to the conclusion that it is more about “quality over quantity”, meaning that the numerical change is not impactful in itself. Rather, it is the change in the score class that matters.

Our study is not exempt from limitations. First, missing hematologic parameters, especially LDH at C2D1, reduced the number of patients that could be tested for paired analyses, as well as the number of overall patients included in the multivariable and stratified regression models accounting for both LIPI at C1D1 and C2D1. We had no possibility of thoroughly assessing molecular features of included tumors and PD-L1 status was mostly unknown. However, most patients were treated in a ≥ 2nd-line setting and in tumors in which PD-L1 status was not mandatory for prescribing the therapy in Europe. Also, the kind of ICI was decided in sponsored trials. Additionally, being a single-center study, our findings require validation in an external cohort. Finally, some patients from the poor LIPI group achieved clinical benefit, possibly because automated neutrophil counts do not discriminate between the different subpopulations of neutrophils that could have protumor or antitumor functions [16, 48, 49]. Besides these limitations, our study confirms the effective prognostic role of baseline LIPI in ICI-treated patients in a pancancer context. LIPI might be especially useful to detect patients more or less likely to derive a clinically meaningful benefit from ICI, potentially serving either as a critical stratification factor or as an inclusion/exclusion criteria in ICI trials and with very limited costs; though further improvements in this research area are advisable. Moreover, early dynamics between C1D1 and C2D1 were able to identify patients with more favorable outcomes beyond baseline score. As far as we are concerned, this is the first study assessing LIPI score early dynamics and its association with survival. Only another study assessed LIPI dynamics, but evaluated the association with side effects and was exclusively conducted in a cohort of patients with NSCLC [50]. Another study from Mezquita et al. was only focused on dNLR, score dynamics were assessed differently, and the setting was restricted to NSCLC [27]*.*These results merit further validation, as LIPI dynamics might become a useful inexpensive on-treatment tool to identify patients either benefiting from continuing immune-checkpoint inhibition or candidates to escalated or alternative therapeutic strategies in the context of adequately designed clinical trials.

## Supplementary Information

Below is the link to the electronic supplementary material.

Supplementary file1 (PDF 913 KB)

## Data Availability

The datasets generated during and/or analyzed during the current study are available from the Corresponding Authors upon reasonable request.
