# Supplementary material for: Blood-based prognostic scores and early dynamics under immunotherapy to select patients with metastatic solid tumors for continuing immune check-point inhibition: a prospective longitudinal study
Source: Cancer Immunol Immunother. 2025 Feb 1;74(3):85. doi: 10.1007/s00262-024-03933-w (PMC11787139; doi:10.1007/s00262-024-03933-w)
Supplement: Supplementary file 1 — Supplementary file1 (PDF 913 KB) [file 262_2024_3933_MOESM1_ESM.pdf]

SUPPLEMENTARY MATERIALS

SUPPLEMENTARY METHODS 2

Statistical analyses – complementary information 2
Supplementary references 2

SUPPLEMENTARY RESULTS 3

AICs of the bivariate Cox and logistic regression models 3

SUPPLEMENTARY FIGURES 5

Supplementary figure 1. Smoothed plots of Schoenfeld residuals for LIPI at C1D1, C2D1 and LIPI shift 5
Supplementary figure 2. Kaplan-Meier curves of OS and PFS according to LIPI score dynamics between C1D1 and C2D1 6

SUPPLEMENTARY TABLES 7

Supplementary table 1. Statistically significant bivariate Cox regressions for the association of prognostic scores with PFS and OS 7
Supplementary table 2. Demographics according to baseline LIPI score 9
Supplementary table 3. Landmark analysis of PFS and OS according to LIPI score classes at different timepoints 12

Supplementary methods

Statistical analyses – complementary information

Variables included in the multivariable logistic regression and in the stratified Cox regression models were the clinical factors that had been found to be differently distributed between RP and NRP. Those variables were the following: treatment type (combination vs. monotherapy; other target vs. anti-PD1/PD-L1), cancer type (non-small cell lung cancer [NSCLC] vs. other), age, ICI treatment line ($\geq 2^{nd}$ vs. 1st), use of systemic antibiotics (ATB) or corticosteroids (>10mg prednisone equivalent dose) during ICI treatment (yes vs. no). Since patients had all been included in clinical trials, ECOG performance status (0-1 vs. >1) was also considered. The proportional hazard assumption regarding the score of interest was previously tested, using correlation coefficients between transformed survival times and scaled Schoenfeld residuals, and further checked with the smoothed plots of Schoenfeld residuals(1), both for OS and PFS. The clinical data cut-off date for this analysis was December 2023. A two-sided alfa error of 0.05 was considered for statistical significance. Considering the observational and exploratory nature of the study, we decided not to take into account the multiplicity issue(2,3).

Supplementary references

1. Schettini F, Conte B, Buono G, et al. T-DM1 versus pertuzumab, trastuzumab and a taxane as first-line therapy of early-relapsed HER2-positive metastatic breast cancer: an Italian multicenter observational study. *ESMO Open*. 2021;6(2):100099. doi:10.1016/j.esmoop.2021.100099

2. Rothman KJ. Six Persistent Research Misconceptions. *J Gen Intern Med*. 2014;29(7):1060-1064. doi:10.1007/s11606-013-2755-z

3. Rothman KJ. No adjustments are needed for multiple comparisons. *Epidemiology*. 1990;1(1):43-46.

Supplementary results

AICs of the bivariate Cox and logistic regression models

AICs of the statistically significant bivariate Cox regression models for PFS (increasing order)

LIPI continuous score model AIC: 1085.70.

RMH continuous score model AIC: 1089.90.

dNLR continuous score model AIC: 1093.07.

RMH categorical score model AIC: 1086.56.

LIPI categorical score model AIC: 1087.59.

AICs of the statistically significant bivariate Cox regression models for OS (increasing order)

LIPI continuous score model AIC: 961.12.

dNLR continuous score model AIC: 968.83.

RMH continuous score model AIC: 973.86.

GRIm continuous score model AIC: 976.05.

PIPO continuous score model AIC: 976.72.

NLR continuous score model AIC: 977.10.

LIPI categorical score model AIC: 962.59.

RMH categorical score model AIC: 968.62.

dNLR categorical score model AIC: 970.40.

NLR categorical score model AIC: 976.78.

PMH categorical score model AIC: 976.69.

PIPO categorical score model AIC: 977.89.

AICs of the statistically significant bivariate logistic regression models for ORR (increasing order)

None.

AICs of the statistically significant bivariate logistic regression models for DCB (increasing order)

RMH categorical score model AIC: 191.83.

AICs of the statistically significant bivariate logistic regression models for rapid PD (increasing order)

LIPI categorical score model AIC: 189.31.

RMH categorical score model AIC: 192.69.

Supplementary figures

Supplementary figure 1. Smoothed plots of Schoenfeld residuals for LIPI at C1D1, C2D1 and LIPI shift

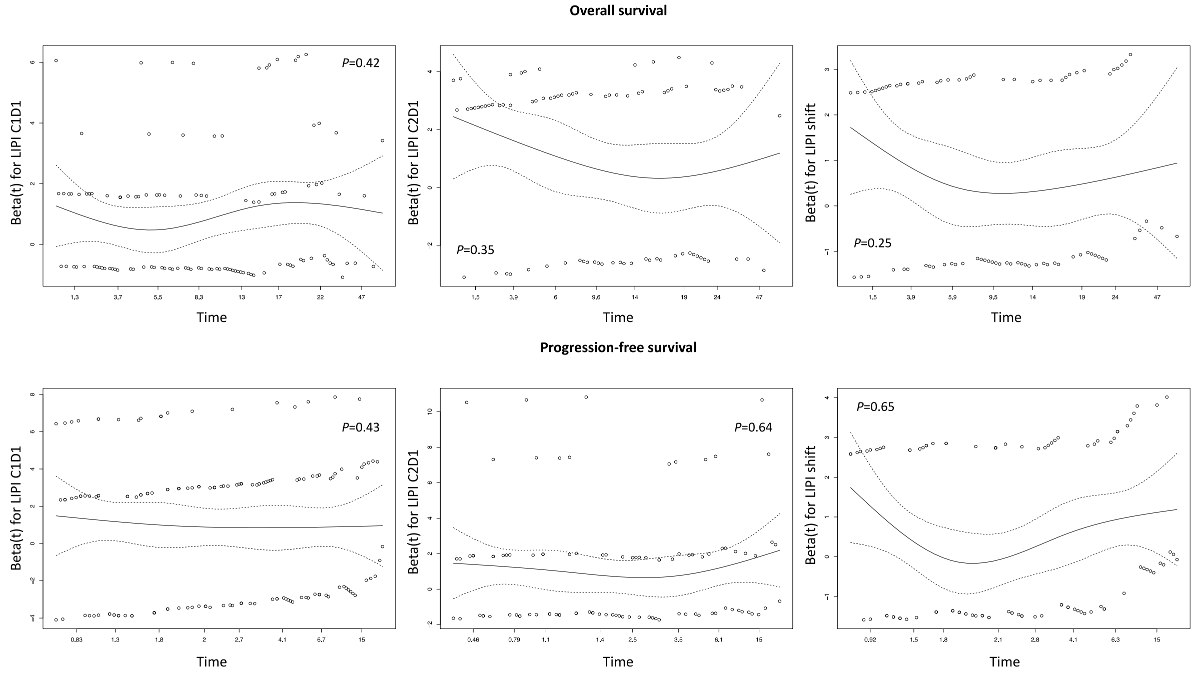

Supplementary figure 2. Kaplan-Meier curves of OS and PFS according to LIPI score dynamics between C1D1 and C2D1

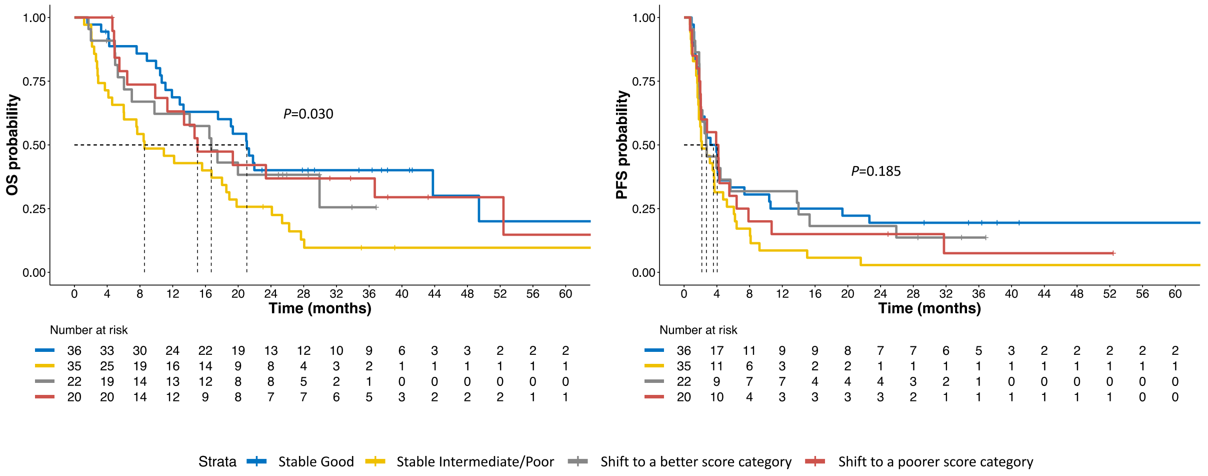

Legend. OS: overall survival; PFS: progression-free survival; C: cycle; D: day. Good, Intermediate and Poor are referred to LIPI score classes.

Supplementary tables

Supplementary table 1. Statistically significant bivariate Cox regressions for the association of prognostic scores with PFS and OS

Prognostic scores	Adj PFS HR*	Inf 95%CI	Sup 95%CI	*P*	Prognostic scores	Adj OS HR*	Inf 95%CI	Sup 95%CI	*P*
LIPI continuous score					*LIPI continuous score*				
	1.67	1.25	2.23	0.001		2.06	1.51	2.82	<0.001
RMH continuous score					*RMH continuous score*				
	1.49	1.12	1.98	0.006		1.54	1.13	2.09	0.005
dNLR continuous score					*GRIm continuous score*				
	1.16	1.02	1.33	0.024		1.50	1.07	2.10	0.006
					NLR continuous score				
-	-	-	-	-		1.08	1.01	1.16	0.025
					dNLR continuous score				
-	-	-	-	-		1.32	1.14	1.51	<0.001
					PIPO continuous score				
-	-	-	-	-		1.24	1.02	1.51	0.030
LIPI				0.002	*LIPI*				<0.001
Intermediate vs. good	1.59	1.07	2.37	0.021	Intermediate vs. good	1.84	1.20	2.84	0.006
Poor vs. good	2.87	1.52	5.42	0.001	Poor vs. good	4.69	2.42	9.09	<0.001
Poor vs. intermediate	1.80	0.97	3.35	0.064	Poor vs. intermediate	2.54	1.35	4.79	0.004
RMH					*RMH*				
Poor vs. good	1.92	1.31	2.82	0.001	Poor vs. good	2.10	1.41	3.15	<0.001
					PMHI				
-	-	-	-	-	Poor vs. good	2.61	1.02	6.68	0.046
					NLR				
-	-	-	-	-	High vs. low	1.65	1.07	2.54	0.023
					dNLR				

-	-	-	-	-	High vs. low	2.31	1.44	3.69	<0.001
					PIPO				0.033
-	-	-	-	-	Intermediate vs. low	0.86	0.55	1.34	0.502
					High vs. low	2.46	1.07	5.67	0.034
-	-	-	-	-	High vs. intermediate	2.87	1.30	6.34	0.009

Legend and footnotes. CI: confidence interval; dNLR: derived NLR; GRIm: Gustave Roussy Immune prognostic score; HR: hazard ratio; Inf: inferior; LIPI: Lung Immune Prognostic Index; NLR: neutrophil-to-lymphocyte ratio; OS: overall survival; PIPO: Phase I Prognostic Online; PMHI: Princess Margaret Hospital Index; PFS: progression-free survival; RMH: Royal Marsden Hospital; Sup: superior. *adjusted for cancer type.

Supplementary table 2. Demographics according to baseline LIPI score

DEMOGRAPHICS		GOOD		INTERMEDIATE		POOR		
		N	%	N	%	N	%	*P*
		73	45.0	68	42.0	21	13.0	
**Age**								
	Median	63.4	-	62.5	-	69.0	-	*0.0076*
	IQR	59.7 - 72.8	-	52.8 - 68.8	-	64.2 - 73.3	-	
**Sex**								
	Male	46	63.0	42	61.8	10	47.6	
	Female	27	37.0	26	38.2	11	52.4	0.4282
	Overall	*73*	*100.0*	*68*	*100.0*	*21*	*100.0*	
**ECOG**								
	0-1	65	89.0	62	91.2	18	85.7	
	≥2	8	11.0	6	8.8	3	14.3	0.7633
	Overall	*73*	*100.0*	*68*	*100.0*	*21*	*100.0*	
**Tumor type**								
	Breast cancer	6	8.2	7	10.3	2	9.5	
	Colorectal adenocarcinoma	10	13.7	17	25.0	5	23.8	
	NSLCL	19	26.0	14	20.6	6	28.6	
	Head and neck	4	5.5	3	4.4	1	4.8	
	Gynecologic tumors (Cervix, endometrium, ovary)	3	4.1	5	7.4	1	4.8	
	Pancreas and biliary tract tumors	3	4.1	3	4.4	0	0.0	
	Esophageal and gastric carcinoma	4	5.5	2	2.9	3	14.3	0.7832
	Melanoma	4	5.5	4	5.9	1	4.8	
	Prostate adenocarcinoma	4	5.5	2	2.9	2	9.5	
	Renal cell carcinoma	2	2.7	4	5.9	0	0.0	
	Urothelial bladder cancer	5	6.8	2	2.9	0	0.0	
	Glioblastoma	6	8.2	2	2.9	0	0.0	
	Other*	3	4.1	3	4.4	0	0.0	
	Overall	*73*	*100.0*	*68*	*100.0*	*21*	*100.0*	
**Number of metastatic sites**								
	<3	20	27.4	13	19.1	0	0.0	
	≥3	53	72.6	55	80.9	21	100.0	*0.0217*
	Overall	*73*	*100.0*	*68*	*100.0*	*21*	*100.0*	
**Metastatic involvement**								
	Visceral	52	71.2	59	86.8	17	81.0	0.0752#
	CNS§	4	5.5	4	5.9	1	4.8	0.9742

Overall	*73*	*100.0*	*68*	*100.0*	*21*	*100.0*	
**RT≤30 days from ICI start**							
Yes	4	5.5	3	4.4	1	4.8	
No	69	94.5	65	95.6	20	95.2	0.9574
Overall	*73*	*100.0*	*68*	100.0	*21*	*100.0*	
**Systemic ATB≤30 days from ICI start**							
Yes	5	6.8	3	4.4	0	0.0	
No	68	93.2	65	95.6	21	100.0	0.4276
Overall	*73*	100.0	*68*	*100.0*	*21*	*100.0*	
**Systemic ATB during ICI**							
Yes	26	35.6	17	25.0	7	33.3	
No	47	64.4	51	75.0	14	66.7	0.3813
Overall	*73*	100.0	*68*	100.0	*21*	*100.0*	
**Systemic corticosteroids ≤30 days from ICI start**							
Yes	11	15.1	7	10.3	4	19.0	
No	62	84.9	61	89.7	17	81.0	0.5225
Overall	*73*	100.0	*68*	100.0	*21*	*100.0*	
**Systemic corticosteroids during ICI**							
Yes	28	38.4	28	41.2	8	38.1	
No	45	61.6	40	58.8	13	61.9	0.9337
Overall	*73*	100.0	*68*	100.0	*21*	*100.0*	
**ICI treatment line**							
1st	20	27.4	15	22.1	4	19.0	
2nd	27	37.0	22	32.4	3	14.3	0.7167
≥3rd	26	35.6	31	45.6	14	66.7	
Overall	*73*	100.0	*68*	100.0	*21*	100.0	
**ICI type**							
Anti-PD1	58	79.5	50	73.5	13	61.9	
Anti-PD-L1	15	20.5	12	17.6	4	19.0	*0.0192*
Other	0	0.0	6	8.8	4	19.0	
Overall	*73*	100.0	*68*	100.0	*21*	100.0	
**Regimen type**							
ICI monotherapy	39	53.4	33	48.5	11	52.4	
ICI combination	9	12.3	21	30.9	4	19.0	0.0802
ICI+other agent	25	34.2	14	20.6	6	28.6	
Overall	*73*	100.0	*68*	100.0	*21*	100.0	
**Previous immunotherapy in every setting**							
Yes	60	82.2	53	77.9	15	71.4	
No	13	17.8	15	22.1	6	28.6	0.5432
Overall	73	*100.0*	68	*100.0*	21	*100.0*	

**PD timing**							
≤4 months from ICI start	35	47.9	49	72.1	15	71.4	
>4 months from ICI start	38	52.1	19	27.9	6	28.6	*0.0079*
Overall	*73*	*100.0*	*68*	*100.0*	*21*	*100.0*	
**Best response**							
CR	3	4.1	2	2.9	0	0.0	
PR	11	15.1	7	10.3	2	9.5	
SD	28	38.4	23	33.8	7	33.3	0.7418
PD	31	42.5	38	55.9	12	57.1	
Overall	*73*	*100.0*	*68*	*100.0*	*21*	*100.0*	

Legend. PS: performance status; IQR: interquartile range; NSCLC: non-small cell lung cancer; ATB: antibiotics; RT: radiotherapy; CNS: central nervous system; ICI: immune-checkpoint inhibitor. *: thymic carcinoma, Merkel cell carcinoma, carcinomas of unknown primary site, soft tissue sarcomas, adrenal gland adenocarcinoma, hepatocarcinoma; #: 2 patients received ICI in 1st or 2nd line, but the precise information was not reported in our records; §: excluding glioblastomas.

Supplementary table 3. Landmark analysis of PFS and OS according to LIPI score classes at different timepoints

TIMEPOINT AND LIPI CLASS			PFS RATES				OS RATES			
**LIPI class**	**N**	**Timepoint**	**N. at risk**	**12-month PFS**	**Inferior 95% CI**	**Superior 95% CI**	**N. at risk**	**12-month OS**	**Inferior 95% CI**	**Superior 95% CI**
Good	73	*C1D1*	17	23.3	15.4	35.3	46	65.0	54.8	77.1
Intermediate	68	*C1D1*	9	13.2	7.2	24.3	31	47.1	36.5	60.8
Poor	21	*C1D1*	2	9.5	2.6	35.6	5	23.8	11.1	51.2
**LIPI class**	**N**	**Timepoint**	**N. at risk**	**24-month PFS**	**Inferior 95% CI**	**Superior 95% CI**	**N. at risk**	**24-month OS**	**Inferior 95% CI**	**Superior 95% CI**
Good	73	*C1D1*	13	17.8	10.9	29.2	24	35.3	25.7	48.4
Intermediate	68	*C1D1*	4	5.9	2.3	15.2	17	27.4	18.5	40.6
Poor	21	*C1D1*	1	4.8	0.7	32.2	2	9.5	2.6	35.6
**TIMEPOINT AND LIPI CLASS**			**PFS RATES**				**OS RATES**			
**LIPI class**	**N**	**Timepoint**	**N. at risk**	**12-month PFS**	**Inferior 95% CI**	**Superior 95% CI**	**N. at risk**	**12-month OS**	**Inferior 95% CI**	**Superior 95% CI**
Good	54	*C2D1*	17	31.5	21.2	46.7	38	73.2	62.1	86.3
Intermediate	48	*C2D1*	6	13.0	6.2	27.5	22	48.2	35.7	65.0
Poor	12	*C2D1*	0	0.0	0.0	0.0	6	50.0	28.4	88.0
**LIPI class**	**N**	**Timepoint**	**N. at risk**	**24-month PFS**	**Inferior 95% CI**	**Superior 95% CI**	**N. at risk**	**24-month OS**	**Inferior 95% CI**	**Superior 95% CI**
Good	54	*C2D1*	12	22.2	13.5	36.6	22	44.4	32.7	60.1
Intermediate	48	*C2D1*	4	8.7	3.4	22.2	13	30.7	19.8	47.4
Poor	12	*C2D1*	0	0.0	0.0	0.0	2	16.7	4.7	59.1

Legend. CI: confidence interval; N. at risk: number of patients alive still at risk of progressing or dying at each given timepoint and according to LIPI score class; PFS: progression-free survival; OS: overall survival; C1D1: day 1 of cycle 1; C2D1: day 1 of cycle 2.
